# Anti-Inflammatory and Neuromodulatory Effects Induced by *Tanacetum parthenium* Water Extract: Results from In Silico, In Vitro and Ex Vivo Studies

**DOI:** 10.3390/molecules26010022

**Published:** 2020-12-23

**Authors:** Lucia Recinella, Annalisa Chiavaroli, Viviana di Giacomo, Marco Daniel Antolini, Alessandra Acquaviva, Sheila Leone, Luigi Brunetti, Luigi Menghini, Gunes Ak, Gokhan Zengin, Simonetta Cristina Di Simone, Claudio Ferrante, Giustino Orlando

**Affiliations:** 1 Department of Pharmacy, Università degli Studi “Gabriele d’Annunzio”, via dei Vestini 31, 66100 Chieti, Italy; lucia.recinella@unich.it (L.R.); annalisa.chiavaroli@unich.it (A.C.); viviana.digiacomo@unich.it (V.d.G.); marcodaniel.antolini@studenti.unich.it (M.D.A.); alessandra.acquaviva@studenti.unich.it (A.A.); sheila.leone@unich.it (S.L.); luigi.brunetti@unich.it (L.B.); luigi.menghini@unich.it (L.M.); disimonesimonetta@gmail.com (S.C.D.S.); giustino.orlando@unich.it (G.O.); 2 Veridia Italia Srl, via Raiale 285, 65100 Pescara, Italy; 3 Department of Biology, Science Faculty, Selcuk University, Campus, 42130 Konya, Turkey; akguneselcuk@gmail.com

**Keywords:** *Tanacetum parthenium*, cortical spreading depression, hypothalamus, interleukins, brain-derived neurotrophic factor, dopamine

## Abstract

*Tanacetum parthenium* (feverfew) has traditionally been employed as a phytotherapeutic remedy in the treatment of migraine. In this study, a commercial *T. parthenium* water extract was investigated to explore its anti-inflammatory and neuromodulatory effects. Isolated mouse cortexes were exposed to a K^+^ 60 mM Krebs-Ringer buffer and treated with *T. parthenium* water extract. The prostaglandin E_2_ (PGE_2_) level, brain-derived neurotrophic factor (BDNF), interleukin-10 (IL-10), and IL-1β gene expression were evaluated in the cortex. The effects on dopamine (DA) release and dopamine transporter (DAT) gene expression were assayed in hypothalamic HypoE22 cells. A bioinformatics analysis was conducted to further investigate the mechanism of action. The extract was effective in reducing cortex PGE_2_ release and IL-1β gene expression. In the same experimental system, IL-10 and BDNF gene expressions increased, and in HypoE22 cells, the extract decreased the extracellular dopamine level and increased the DAT gene expression due to the direct interaction of parthenolide with the DAT. Overall, the present findings highlight the efficacy of *T. parthenium* water extract in controlling the inflammatory pathways that occur during cortical-spreading depression. Additionally, the inhibition of the hypothalamic DA release observed in this study further supports the role of dopaminergic pathways as key targets for novel pharmacological approaches in the management of migraine attacks.

## 1. Introduction

Migraine is one of the most prevalent neurovascular disorders, the incidence of which ranges from 8% to 14.7% of people. The disease begins in childhood, with a tendency to worsen during adulthood (22–55 years of age). Women experience a higher incidence compared to men (3:1) [1]. Serotonin (5-HT) depletion and trigemino-vascular system activation are notoriously involved in migraine pathophysiology [2,3,4,5,6], although the origin site and mechanisms at the basis of migraine are still a matter of debate. In this context, cortical spreading depression (CSD), a supraphysiological and neurotoxic depolarizing stimulus, has been described as a putative link between 5-HT depletion and trigeminal nociception [7]. The involvement of the hypothalamus in the complex pathophysiological events underlying migraine attacks has also been suggested [8]. While it also plays a key role in regulating hormone release and energy balance [9], the hypothalamus is anatomically connected with the brain’s pain-modulating system and trigeminal nuclei, which regulate nociception [8]. Hypothalamic orexigenic factors, specifically orexins and dopamine (DA), are possibly involved both in CSD and migraine [10,11,12]. In this context, the use of metoclopramide, a DA receptor antagonist, in controlling the clinical symptoms related to migraine [13] is sensible, although the recommended first-line treatments for mild to moderate forms of migraine are non-steroidal anti-inflammatory drugs and acetaminophen, whereas triptans are recommended in more severe migraine attacks [14]. In contrast, antiepileptics, antidepressants, and antihypertensives are considered first-line options for preventing the attacks [15]. However, a new approach to migraine treatment has arisen from the use of the anti-CGRP monoclonal antibody erenumab, which demonstrated efficacy, tolerability, and good compliance [16]. Despite the numerous synthetic drugs available for the treatment of migraine, the incidence of severe adverse effects [17] has led to the search for new therapeutic options, including herbal formulations and nutraceuticals that maintain acceptable levels of efficacy but have minor side effects [18]. *Tanacetum parthenium* (feverfew), which belongs to the Asteraceae family, has been traditionally employed as a phytotherapeutic remedy in the treatment of migraine [19,20]. This use is consistent with the intrinsic antioxidant and anti-inflammatory properties of the active components of the phytocomplex, including flavonoids, volatile oils, and parthenolide [21,22]. Recently, the neuromodulatory effects of *T. parthenium* were described, particularly in the inhibition of 5-HT turnover in a preclinical ex vivo model of CSD [23]. Additionally, parthenolide was reported to modulate DA release in the ventral tegmental area [24]. However, there is still lack of scientific literature regarding anti-inflammatory and neuromodulatory effects induced by *T. parthenium* in the brain, with no studies conducted on hypothalamic pathways.

The aim of this study was to further explain the anti-inflammatory effects of a water extract of *T. parthenium*. Specifically, the gene expression of interleukin (IL)-1β, the IL-10 and brain-derived neurotrophic factor (BDNF), and the release of prostaglandin E_2_ (PGE_2_) were measured in isolated cortex specimens perfused with a K^+^ 60 mM Krebs-Ringer buffer employed as a supraphysiological depolarizing and excitotoxic stimulus. This ex vivo model was selected to reproduce CSD [23]. Additionally, the modulatory effects of *T. parthenium* on DA release and the gene expression of the DA transporter (DAT) were investigated in hypothalamic HypoE22 cells. A qualitative mass spectrometry (MS) analysis was also conducted to explore the phytochemical composition of the extract. The identified phytocompounds were then analyzed using STITCH (http://stitch.embl.de), a bioinformatic software platform for plotting a targets–components analysis to predict, albeit partially, the mechanism of the action underlying the observed biopharmacological effects.

## 2. Results and Discussion

**Figure 1:**
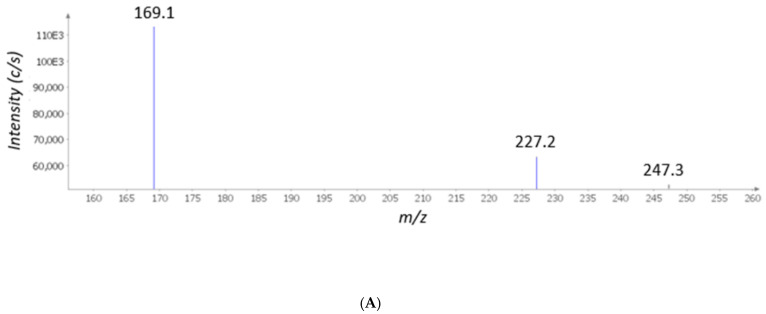

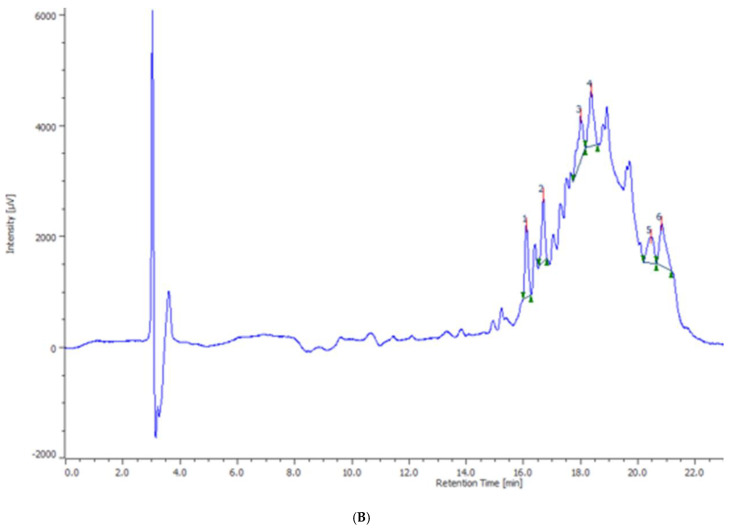
(**A**) The qualitative mass spectrometry (MS) analysis conducted on the *Tanacetum parthenium* water extract. The MS analysis of gallic acid, resveratrol, and parthenolide was conducted in negative ion mode (*m*/*z* scan mode: 119–556), and the *m*/*z* ratios of 169.1, 227.2, and 247.3 were monitored to identify gallic acid, resveratrol, and parthenolide, respectively. (**B**) The qualitative the high performance liquid chromatography coupled to diode-array detection (HPLC-DAD) analysis showing the presence of catechin (1), chlorogenic acid (2), and rutin (4) in the *T. parthenium* extract. The chromatographic analysis also showed the presence of unidentified phenolic compounds (3, 5, 6).

Initially, the extract was studied phytochemically through the MS technique. The MS qualitative investigation focused on the presence of parthenolide, the characterizing phytochemical of *T. parthenium*, and gallic acid and resveratrol, which were previously measured in the extract with independent HPLC-fluorimetric analysis [23]. In accordance with our recent studies [25,26], the MS analysis of gallic acid, resveratrol, and parthenolide was conducted in negative ion mode (*m*/*z* scan mode: 119–556), and the *m*/*z* ratios of 169.1, 227.2, and 247.3 were monitored to identify gallic acid, resveratrol, and parthenolide, respectively (Figure 1A). In our previous study, catechins were also detected in the extract, although at lower concentrations (0.11–0.23% *w*/*w* dry extract) compared to gallic acid (72.47% *w*/*w* dry extract) and resveratrol (0.79% *w*/*w* dry extract) [23]. The high intensity of the MS signal measured for gallic acid further supported our previous investigation, which showed gallic acid as the most prominent compound in the *T. parthenium* extract [23].

**Figure 2:**
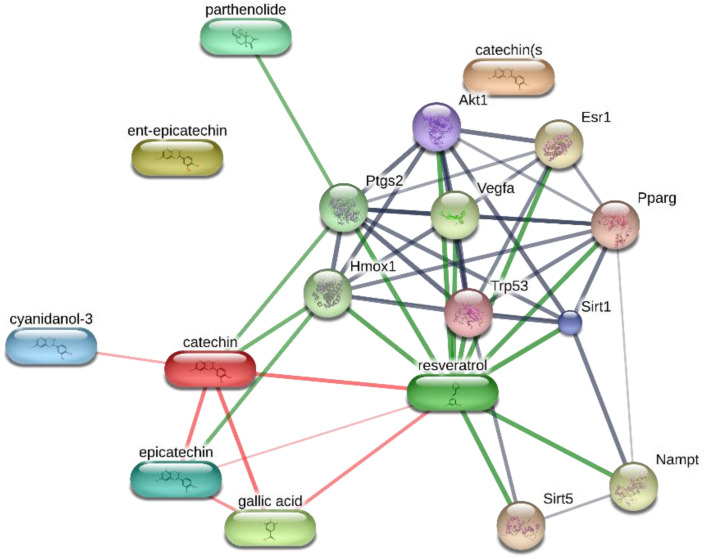
The targets–components analysis conducted via the STITCH platform (http://stitch.embl.de), highlighting the prominent role of cyclooxygenase-2 (COX-2/Ptgs2) in the components–targets plot.

**Figure 3:**
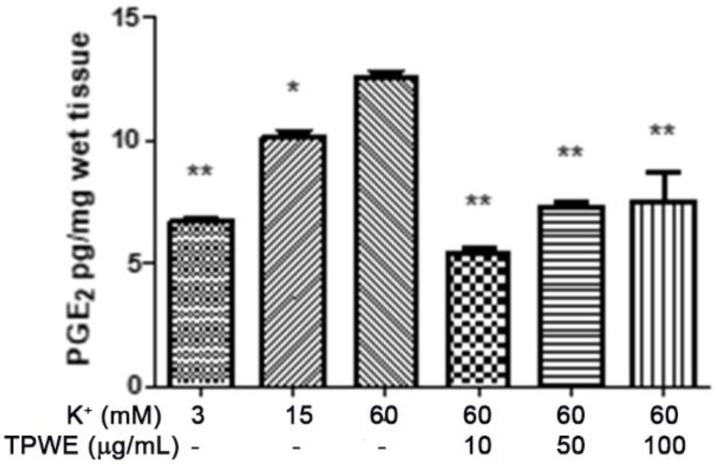
The inhibitory effect induced by *Tanacetum parthenium* water extract (TPWE: 10–100 µg/mL) on prostaglandin E_2_ (PGE_2_) level (pg/mg wet tissue) in isolated cortexes challenged with K^+^ 60 mM. ANOVA, *p* < 0.01; * *p* < 0.05, ** *p* < 0.01 vs. the K^+^ 60 mM group.

The extract was also qualitatively analyzed using the high performance liquid chromatography coupled to diode-array detection (HPLC-DAD) technique, which revealed the presence of other phenolic compounds in the extract, including catechin (1), chlorogenic acid (2), and rutin (4) (Figure 1B); while the phenolic compounds 3, 5, and 6 were not identified. Previous studies have also highlighted the presence of resveratrol in *T. parthenium*. Resveratrol is present in many plant families and species, leading to an increased interest in finding the compound in plant extracts [27]. This interest is related to the downregulating effects of resveratrol on oxidative and inflammatory pathways [28]. In a preliminary study, we also demonstrated the efficacy of resveratrol in reducing the ex vivo hydrogen-peroxide-induced lipid peroxidation in cortex synaptosomes [29]. The results of the phytochemical analysis were crucial for conducting the preclinical study to determine the efficacy of the *T. parthenium* water extract in controlling the clinical symptoms related to migraine, particularly the inflammatory pathways related to CSD and the DA signaling at the hypothalamic level. Using the phytochemical composition of the extract, a targets–components analysis was carried out to predict putative targets underlying the antioxidant and anti-inflammatory effects on the brain. The STITCH platform was used to predict direct interactions of gallic acid, resveratrol, catechin, and parthenolide with different proteins involved in inflammatory response (Figure 2), although cyclooxygenase-2 (COX-2), which was found to be up-regulated in experimental models of CSD [30], seemed to play a prominent role in the targets–components analysis. The release of PGE_2_, the major product and biomarker of COX-2 activity, was measured in isolated cortexes exposed to K^+^ 60 mM and treated with the *T. parthenium* extract (10–100 µg/mL). The concentration range was selected basing on our previous study [23]. The blunting effect induced by the extract on the K^+^ 60 mM-induced PGE_2_ level (Figure 3) supports the hypothesis that a potential modulation of the inflammatory component characterizes CSD [31].

**Figure 4:**
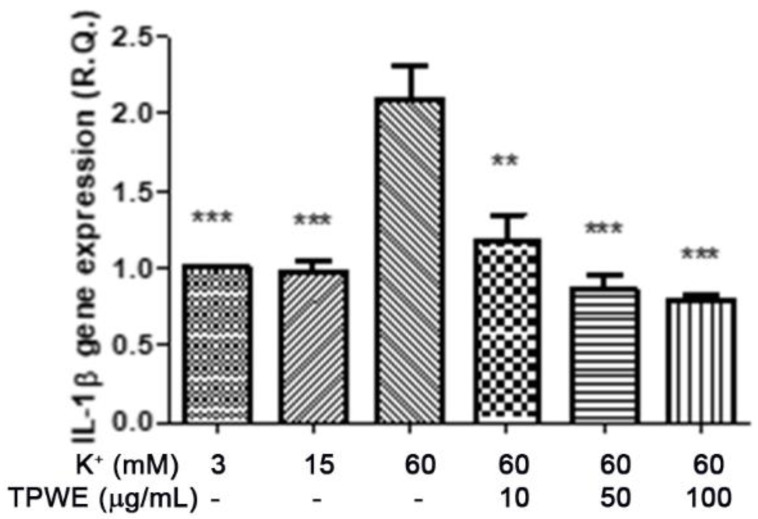
The inhibitory effect induced by the *Tanacetum parthenium* water extract (TPWE: 10–100 µg/mL) on interleukin-1β (IL-1β) gene expression (R.Q. = relative quantification) in isolated cortexes challenged with K^+^ 60 mM. ANOVA, *p* < 0.0001; ** *p* < 0.01, *** *p* < 0.001 vs. the K^+^ 60 mM group.

**Figure 5:**
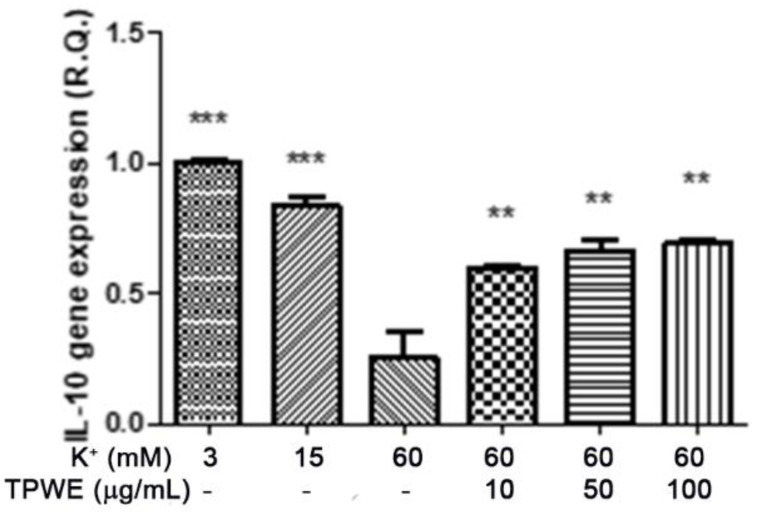
The stimulating effect induced by *Tanacetum parthenium* water extract (TPWE: 10–100 µg/mL) on interleukin-10 (IL-10) gene expression (R.Q. = relative quantification) in isolated cortexes challenged with K^+^ 60 mM. ANOVA, *p* < 0.0001; ** *p* < 0.01, *** *p* < 0.001 vs. the K^+^ 60 mM group.

**Figure 6:**
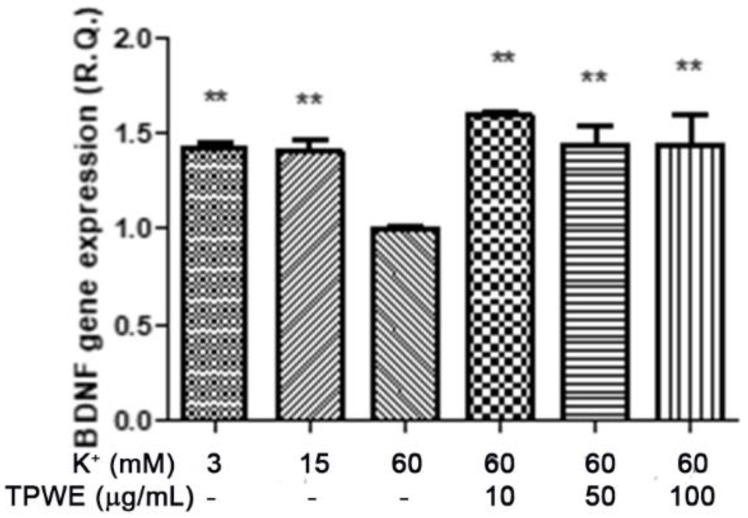
The stimulating effect induced by *Tanacetum parthenium* water extract (TPWE: 10–100 µg/mL) on the brain-derived neurotrophic factor (BDNF) gene expression (R.Q. = relative quantification) in isolated cortexes challenged with K^+^ 60 mM. ANOVA, *p* < 0.0001; ** *p* < 0.01 vs. the K^+^ 60 mM group.

**Figure 7:**
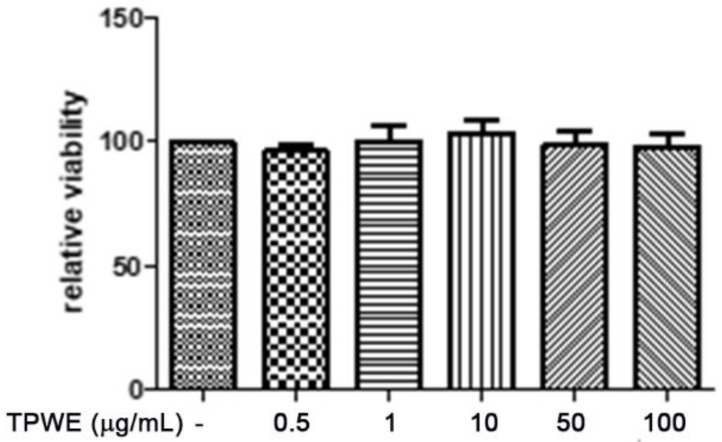
The null effect induced by *Tanacetum parthenium* (TPWE: 0.5–100 µg/mL) on the viability of the hypothalamic HypoE22 cell line evaluated using an MTT test.

**Figure 8:**
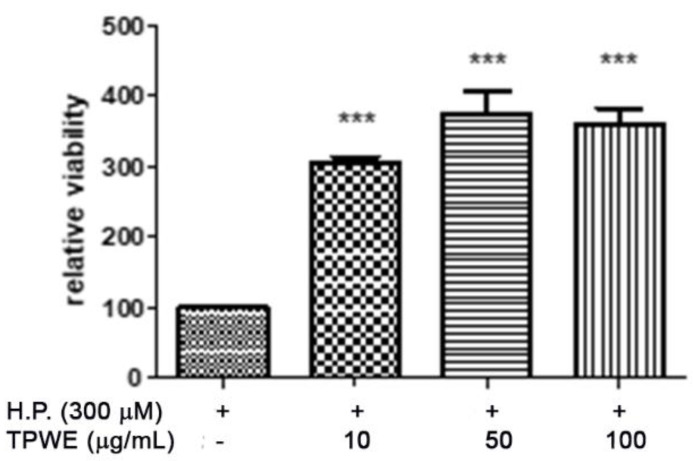
The stimulating effect induced by the *Tanacetum parthenium* water extract (TPWE: 10–100 µg/mL) on the viability of HypoE22 cells exposed to 300 µM of hydrogen peroxide (H.P.). The cell viability was evaluated using an MTT test. ANOVA, *p* < 0.0001, *** *p* < 0.001 vs. the H.P. group.

**Figure 9:**
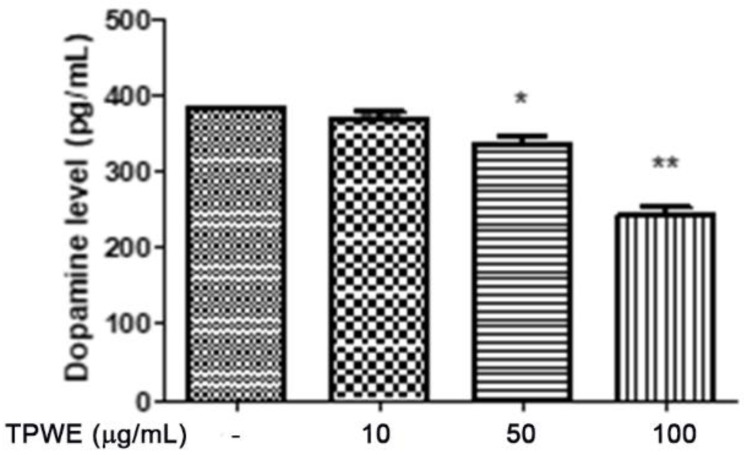
The inhibitory effect induced by *Tanacetum parthenium* (TPWE: 10–100 µg/mL) on dopamine release (pg/mL) from hypothalamic HypoE22 cells. ANOVA, *p* < 0.001; * *p* < 0.05, ** *p* < 0.01 vs. the untreated control (CTR) group.

**Figure 10:**
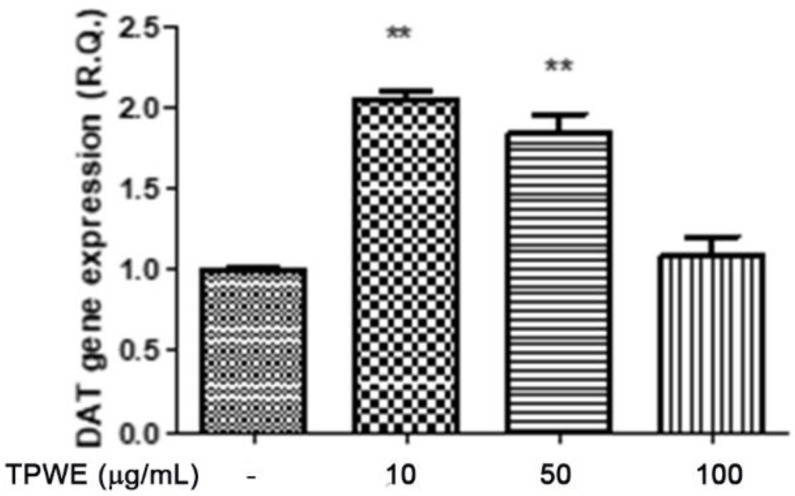
The inhibitory effect induced by *Tanacetum parthenium* (TPWE: 10–100 µg/mL) on dopamine transporter (DAT) gene expression in hypothalamic HypoE22 cells. ANOVA, *p* < 0.001; ** *p* < 0.01 vs. the untreated control (CTR) group.

**Figure 11:**
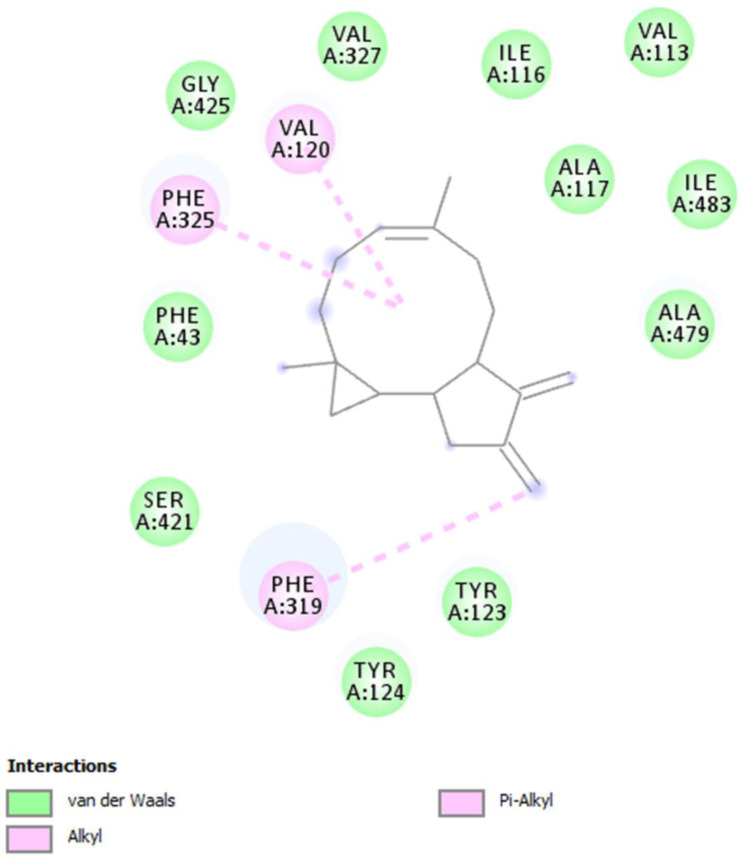

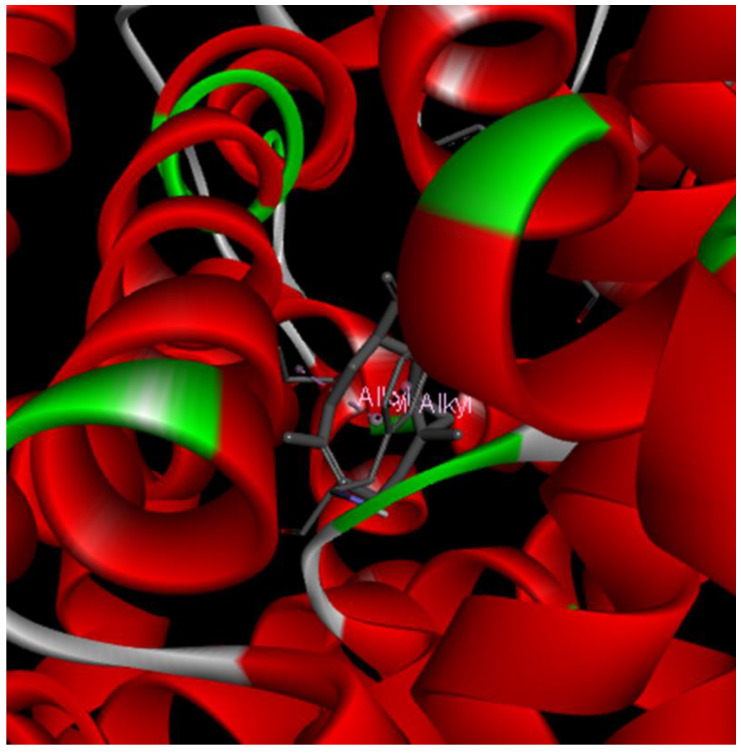
The putative interactions between parthenolide and the dopamine transporter [DAT; Protein Data Bank ID (PDB): 4XNX_1]. The free energy of binding (ΔG) and affinity (Ki) were −8.6 kcal/mol and 0.6 µM, respectively.

To confirm this modulation, the gene expressions of pro-inflammatory IL-1β and anti-inflammatory IL-10 were evaluated (Figure 4 and Figure 5), and the extract was found to have exerted a significant pro-homeostatic effect on both cytokines. These results also bolster our previous observations of reduced oxidative stress and serotonin turnover induced by the extract in isolated cortexes [23], which could be related, albeit partially, to the intrinsic scavenging and reducing effects of gallic acid, the most abundant compound in the extract [32]. Materazzi et al. showed that parthenolide could also target the ankyrin 1 (TRPA1) channel, and this interaction could partly mediate the *T. parthenium* anti-migraine effect [22]. The activation of the TRPA1 receptor was also related to anti-inflammatory effects, as evidenced by reduced in vivo COX-2 activity and IL-1β level [33]. In our study, we also investigated the extract’s effect on the gene expression of BDNF, and found that the extract exerted a blunting effect on the K^+^ 60 mM-induced reduction of BDNF mRNA levels (Figure 6). Neurotrophins, including BDNF, were related to chronic and episodic migraine, and the plasma levels of this peptide were found to be lower in the migraineurs compared to the control group [34]. The authors of that study also suggested that the reduction of BDNF plasma levels could be related, at least partially, to the hyperactivation of the hypothalamic–pituitary axis that occurs during a migraine attack [35]. It has been proposed that the hypothalamus plays a pivotal role in the onset of a migraine attack [8]. In this context, the attention of researchers has been focused on the involvement of hypothalamic orexigenic factors, including orexins and dopamine. The pharmacological blockade of orexin receptors was found to be effective in counteracting CSD in rats [10], while the stimulation of hypothalamic dopamine signaling is reputed to mediate yawning, fatigue, and alterations in nausea and appetite [11,12]. Additionally, hypothalamic neurons containing DA and orexins are connected to trigemino-vascular thalamic neurons that project to the cortex, thus further highlighting the role of the hypothalamus in the onset of a migraine attack [36,37]. Therefore, the effects of the *T. parthenium* water extract were evaluated on hypothalamic HypoE22 cells. Initially, the biocompatibility of the extract was investigated in the concentration range of 0.5–100 µg/mL. A null effect on cell viability was found in basal conditions, while a stimulation of the viability of the cells exposed to 300 µM hydrogen peroxide (Figure 7 and Figure 8) was registered, which further supports the observed protective effects in the cortex. Additionally, the extracellular level of DA was evaluated in cells exposed to the extract at 10–100 µg/mL, and found a concentration-dependent reduction of neurotransmitter release from the hypothalamic cells (Figure 9). We hypothesize that this effect could be related, at least in part, to the presence of parthenolide in the extract. This phytocompound was found to be effective in reducing the spontaneous DA firing in the ventral tegmental area in rats [24]. The same authors also proposed that the inhibition of DA release induced by parthenolide could occur through binding at the membrane monoamine transporters. To explore the mechanism at the basis of the reduced DA level in HypoE22 cells treated with the extract, the gene expression of dopamine transporter (DAT) was measured. The DAT gene expression was significantly up-regulated at the lowest tested concentrations of 10–50 µg/mL (Figure 10), thus indicating a direct modulatory effect of DAT underlying the reduced release of DA from hypothalamic cells. This result is also supported by the docking run, which showed putative interactions of parthenolide with the DAT binding site at sub-micromolar concentration (Ki = 0.6 µM) (Figure 11).

## 3. Materials and Methods

### 3.1. Extract Preparation

The commercial *T. parthenium* water extract containing 0.5% parthenolide was provided as dried material by Cristalfarma S.r.l. (Milan, Italy). The extract was rehydrated in a Trans-sonic T460 ultrasonic bath (Elma, Singen, Germany) for 10 min at room temperature and full power (35 kHz), as previously described [38].

### 3.2. Phytochemical Analysis

The *T. parthenium* extract was analyzed qualitatively using an expression compact mass spectrometer (CMS) (Advion, Ithaca, NY, USA) in negative ion mode (*m*/*z* scan mode: 119–556). The signal identification of gallic acid, resveratrol, and parthenolide was realized as previously described [25,26]. The qualitative characterization of the extract also included HPLC-DAD analysis. The HPLC apparatus consisted of a PU-2080 PLUS chromatographic pump, a DG-2080-54 line degasser, a mix-2080-32 mixer, a diode array detector (DAD), an AS-2057 PLUS autosampler, and a CO-2060 PLUS column thermostat (all from Jasco, Tokyo, Japan). The integration was performed using ChromNAV2 Chromatography software. The gradient elution (water–acetonitrile: 93:7 ratio) was conducted according to previous literature [39]. The separation was performed on an Infinity lab Poroshell 120 reverse phase column (C18, 150 mm × 4.6 mm i.d., 2.7 µm) (Agilent Santa Clara, CA, USA). The column temperature was set to 30 °C.

### 3.3. In Vitro Studies

The HypoE22 rat-hypothalamus cell line was purchased from Cedarlane Corporation (Burlington, ON, Canada) and cultured in Dulbecco’s Modified Eagle Medium (DMEM) supplemented with 10% (*v*/*v*) heat-inactivated fetal bovine serum and penicillin–streptomycin (100 μg/mL) (all from EuroClone SpA Life-Sciences-Division, Milano, Italy). The cells were grown at 37 °C in a humidified atmosphere of 5% CO_2_. When indicated, the cells were treated with H_2_O_2_ (300 μM) for 3 h and with different concentrations of *T. parthenium* extract (10–100 µg/mL). The cell viability was evaluated after 24 h of culturing using the MTT (3‒[4,5‒dimethyl‒thiazol‒2‒yl‒]‒2,5‒diphenyl tetrazolium bromide) growth assay (Sigma-Aldrich, St. Louis, MO, USA), based on the capability of viable cells to reduce MTT to a colored formazan product. Details about the protocol were reported in our recent study [40].

### 3.4. Ex Vivo CSD Paradigm

The study included 24 adult male C57BL6 mice housed in Plexiglass cages (40 cm × 25 cm × 15 cm), with two mice per cage, in climatized colony rooms (22 ± 1 °C; 60% humidity), on a 12 h/12 h light/dark cycle (light phase: 07:00–19:00 h), with free access to tap water and food 24 h/day throughout the study, with no fasting periods. The mice were fed a standard laboratory diet (3.5% fat, 63% carbohydrate, 14% protein, and 19.5% other components without a caloric value; 3.20 kcal/g). The housing conditions and experimentation procedures were strictly in accordance with the European Union’s ethical regulations on the care of animals for scientific research. The experiments were approved by the Local Ethical Committee (University “G. d’Annunzio” of Chieti-Pescara) and the Italian Health Ministry (authorization number: F4738.N.5QP). The mice were euthanized via CO_2_ inhalation (100% CO_2_ at a flow rate of 20% of the chamber volume per min), and the cortex specimens were immediately collected and maintained in a thermostatic shaking bath at 37 °C for an incubation period of 1 h in a Krebs-Ringer buffer at different K^+^ concentrations, as described below:

1. K^+^ 3 mM, corresponding to the basal condition;
2. K^+^ 15 mM, corresponding to the physiologic depolarizing stimulus;
3. K^+^ 60 mM, corresponding to the excitotoxic depolarizing stimulus.

The experiment, which reproduced the neural pathophysiological condition known as CSD, was designed according to previous ex vivo and in vivo studies that described the use of elevated K^+^ concentrations (up to 50–60 mM) to induce injury to the central nervous system [41]. During incubation, the cortex specimens were exposed to *T. parthenium* (1–100 μg/mL). Finally, the tissue perfusates were collected, and the PGE_2_ levels (ng/mg wet tissue) were measured using a radioimmunoassay (RIA), as previously reported [42]. Specific anti-8-iso-PGF_2α_ and anti-PGE_2_ were developed in a rabbit; the cross-reactivity against other prostanoids was <0.3%. A sample of 100 μL of prostaglandin was incubated overnight at 4 °C with the ^3^H-prostaglandin (3000 cpm/tube) and an antibody (final dilution: 1:120,000; provided by Professor G. Ciabattoni), in a volume of 1.5 mL of 0.025 M phosphate buffer. The free and antibody-bound prostaglandins were separated by the addition of 100 μL of 5% bovine serum albumin and 100 μL of 3% charcoal suspension, centrifuged for 10 min at 4000× *g* at 5 °C, and decanted off the supernatants into a scintillation fluid (Ultima Gold™, Perkin Elmer, Waltham, MA, USA) for β-emission counting. The detection limit of the assay method was 0.6 pg/mL.

### 3.5. RNA Extraction, Reverse Transcription, and Real-Time Reverse Transcription Polymerase Chain Reaction (Real-Time RT PCR)

The total RNA was extracted from the mouse cortex specimens and rat hypothalamic HypoE22 cells using TRI Reagent (Sigma-Aldrich, St. Louis, MO, USA), according to the manufacturer’s protocol. Contaminating DNA was removed using 2 units of RNase-free DNase 1 (DNA-free kit, Ambion, Austin, TX, USA). The RNA concentration was quantified at 260 nm using a spectrophotometer reading (BioPhotometer, Eppendorf, Hamburg, Germany), and its purity was assessed using the ratio read at 260 and 280 nm. The quality of the extracted RNA samples was also determined using electrophoresis through agarose gels and staining with ethidium bromide under UV light. One microgram of total RNA extracted from each sample in a 20 µL reaction volume was reverse transcribed using a High-Capacity cDNA Reverse Transcription Kit (Thermo Fisher Scientific Inc., Monza, Italy). The reactions were incubated in a 2720 Thermal Cycler (Thermo Fisher Scientific Inc., Monza, Italy) initially at 25 °C for 10 min, then at 37 °C for 120 min, and finally at 85 °C for 5 s. The gene expressions of IL-1β, IL-10, and BDNF in mouse cortex specimens, and DAT in HypoE22 cells, were determined via quantitative real-time PCR using TaqMan probe-based chemistry, as previously described [43]. The PCR primers and TaqMan probes, including β-actin used as the housekeeping gene, were purchased from Thermo Fisher Scientific Inc. The Assays-on-Demand Gene Expression Products used for gene-expression evaluations in the mouse cortex specimens were: Mm00434228_m1 for the IL-1β gene, Mm01288386_m1 for the IL-10 gene, Mm04230607_s1 for the BDNF gene, and Mm00607939_s1 for the β-actin gene. The Assays-on-Demand Gene Expression Products used for gene-expression evaluations in the rat-hypothalamic HypoE22 cells were: Rn00562224_m1 for the DAT gene, and Rn00667869_m1 for the ACTB gene. The real-time PCR was carried out in triplicate for each cDNA sample for each of the investigated genes. The data were elaborated with the Sequence Detection System (SDS), software version 2.3 (Thermo Fisher Scientific Inc.). The gene expression was relatively quantified using the comparative 2^−∆∆Ct^ method [44].

### 3.6. High-Performance Liquid Chromatography (HPLC) Determination of DA

The extracellular DA levels were analyzed using an HPLC apparatus consisting of a PU-2080 chromatographic pump (Jasco, Tokyo, Japan) and an ESA Coulochem III coulometric detector (Chelmsford, MA, USA) equipped with a microdialysis cell (ESA-5014b) with a porous graphite working electrode and a solid-state palladium reference electrode. The analytical conditions for biogenic amine identification and quantification were selected in accordance with our previous study [45].

### 3.7. Bioinformatics

The chemical structures were prepared and converted to mol files using ChemSketch software. A compound-target analysis was also conducted using the bioinformatics platform STITCH to predict putative targets. The routine steps in the calculations for the docking analysis involved the preparation of the inhibitors and the protein. The crystal structures of the protein were downloaded from the Protein Data Bank (PDB; https://www.rcsb.org). The PDB code was 4XNX_1 (dopamine transporter: DAT). To prepare the protein for docking calculations, all water molecules and co-crystalized compounds were removed, and polar hydrogen atoms and neutralization were added using the Autodock4 program (Molinspiration database). The starting structures of the secondary metabolites were optimized to their ground-state structures using the Austin Model 1 (AM1) semi-empirical method, and the 3D structures were saved in the mol2 file format. The protein was immersed in a 3D grid box with dimensions of 60 × 60 × 60 and a distance between points of 0.375 Å. The Lamarckian genetic algorithm was used to calculate the docking free energy of 250 conformations for each inhibitor. The docking results were clustered and organized according to the docking free energy. The binding site was localized, and the nonbonding interactions were elucidated using the Discovery Studio 5.0 visualizer.

### 3.8. Statistical Analysis

The GraphPad Prism for Windows v5.01 (GraphPad Software, San Diego, CA, USA) was used to analyze the experimental results. The means ± SD were determined for each experimental group and analyzed using one-way analysis of variance (ANOVA), followed by a Newman–Keuls comparison multiple test. The statistical significance was set at *p* < 0.05. The number of animals to be employed in the study was calculated using G*Power software (v3.1.9.4). The values of the study potency (1-β) and the significance level (α) were 0.8 and 0.05, respectively.

## 4. Conclusions

The findings of our study highlight the efficacy of *T. parthenium* water extract in controlling the inflammatory pathways that occur during CSD. Additionally, the inhibition of hypothalamic DA release observed in our study further supports the dopaminergic pathways as key targets for novel pharmacological approaches in counteracting migraine attacks. Further studies are needed to confirm the present findings with in vivo models.

## Data Availability

The data presented in this study are available on request from the corresponding author.

## Sample Availability

Samples of the compounds are available from the authors.
